# Performance Profiling—Perspectives for Anti-doping and beyond

**DOI:** 10.3389/fphys.2017.01102

**Published:** 2017-12-22

**Authors:** Sergei Iljukov, Yorck O. Schumacher

**Affiliations:** ^1^KIHU - Research Institute for Olympic Sports, Jyvaskyla, Finland; ^2^Aspetar Orthopaedic and Sports Medicine Hospital, Doha, Qatar

**Keywords:** competition results, analysis, passport, monitoring, match fixing, target testing

## Abstract

Performance profiling is a new area of research that could potentially open new frontiers in the fight against doping. Even beyond exposing unnatural and pharmacology aided performances, there are other potential applications and benefits of performance modeling for the protection of the integrity of sports. The backbone of performance modeling in anti-doping is the individual tracking of performance through competition results or other metrics of sporting achievements. Since performance improvement is the primary goal of doping, it is expected that doping will affect competition results. Thus, individual tracking of performance could potentially expose suspicious cases that deserve more scrutiny from anti-doping officials and help to adjust targeted testing. On the other hand changes in performance levels could also be used to assess the efficiency of new anti-doping strategies. Another application of performance analysis is to develop unified classifications of athletes according to their level of performance. This classification has numerous practical meanings, but from anti-doping perspective it provides an opportunity to set exact criteria for athletes belonging to national and international testing pools and thus estimate the number of tests needed in different countries based on the number of athletes at ascertain performance level. At the moment, in the absence of unified and comprehensive criteria for national and international testing pools, there are no definitive regulations regarding exact doping test numbers needed. Thus, it creates inequality between nations and affects the credibility of the anti-doping system worldwide. Such classification would allow a more efficient use of anti-doping resources. Since doping is not the only threat to the integrity of sports, performance modeling can also help to reveal cases of other misbehavior in sports, like match fixing or result manipulation. In summary, performance modeling and its application to various fields is a new method to improve the efficiency of systems to safeguard the integrity of sports at different levels.

## Introduction

The true prevalence of doping use in elite sports is unknown, but studies give estimates between 14 and 39% (de Hon et al., 2015). According to WADA statistics, ~2% of the collected doping samples yearly are reported to contain a banned substance (WADA Anti-Doping Testing Figures, 2015). The figure remains quite stable despite the gradual increase of both the number of doping tests conducted and the sensitivity of the analytical methods. Based on these findings, a substantial discrepancy between the estimated prevalence of doping and the number of positive doping cases remains. This fact reasonably raises questions about current efficiency of anti-doping testing strategies.

The key aim of any doping technique is to improve sporting performance. It can therefore be assumed that tracking individual performance data could reveal suspicious and disproportionate performance gains in doping athletes, which might differentiate them from their undoped peers and thus help to use existing anti-doping resources more efficiently. The idea of performance tracking for anti-doping purposes was originally presented by Schumacher and Pottgiesser (2009). Developing this new concept, the purpose of this publication is to provide a broad perspective on potential performance profiling applications in anti-doping and other forms of cheating in sports.

## Conceptual thoughts

Of course, improvement of athletic performance *per se* is not an indication of any wrongdoing and must be interpreted with caution. The key problem in the longitudinal tracking of performance is therefore to differentiate between a physiological increase in performance caused by training and/or maturation and an unphysiological improvement caused by doping. There are currently no international benchmarks and very little scientific research on the physiological improvement in performance and its variation in a longitudinal setting, which could be used for this purpose.

Nevertheless, two basic approaches to achieve a qualitative weighing of performances appear practicable in various combinations: the comparison of the athlete performances in absolute terms or the comparison of an athlete's performance with his peers. While the first approach is easily conceivable in measurable sports, such as “centimeter-grams-seconds” (CGS) disciplines, where the performance can be expressed as a measurable number, the second approach is suited for match sports, where the performance is measured as a win or loss in a head to head confrontation with a peer athlete (f.ex. ILO classification in chess). Of course, combinations of both methods are conceivable: for example, a certain time over 100 m would classify an athlete in the Xth percentile in his age group. A subsequent improvement of his time would then possibly be compared in absolute terms to his previous results and to his percentile ranking within his peers.

Considering such approaches, different applications in the realm of anti-doping become conceivable, which are briefly illustrated below.

## Increasing the efficiency of anti-doping measures by adjusting targeted testing using performance data

While a very rudimentary form of performance based anti-doping testing is already used in all sports, where the winners or podium placings of competitions usually have to provide anti-doping samples, such approach can be fine-tuned further by including a broader base of information.

In a case report, we demonstrated the feasibility of this method (Iljukov et al., under review). Historical competitive data of an athlete were used to calculate theoretical future performances for given running distances based on existing and validated critical power models. When these projected performances were significantly exceeded, a dedicated target testing scheme through the athlete biological passport (ABP) was initiated, based on which the athlete was subsequently found guilty of blood manipulation. Such tracking and classification of performances could be used for target testing purposes and improve the in competition testing, where usually only winners/place getters and a few random athletes are tested.

As mentioned above, benchmarks for performance development and variability are still scarce and make interpretation of individual performance changes somehow arbitrary. First scientific results however indicate that the individual variation of performance in a given event and over relatively short timeframes is limited (Hopkins and Hewson, 2001). To make the approach more practicable, more data on the longitudinal development over longer time frames is needed. The growing databases of competition results in all sports for many different age groups and the ability to process large amounts of data electronically might help with this problem. In fact, from the amount of data available, benchmarks for normal performance development and variability profiles might already be derived. A potential drawback is the presence of doped athletes in such databases, which might bias the results.

## Performance profiling to indicate the introduction of new doping substances or to evaluate the efficiency of anti-doping strategies

**Figure 1:**
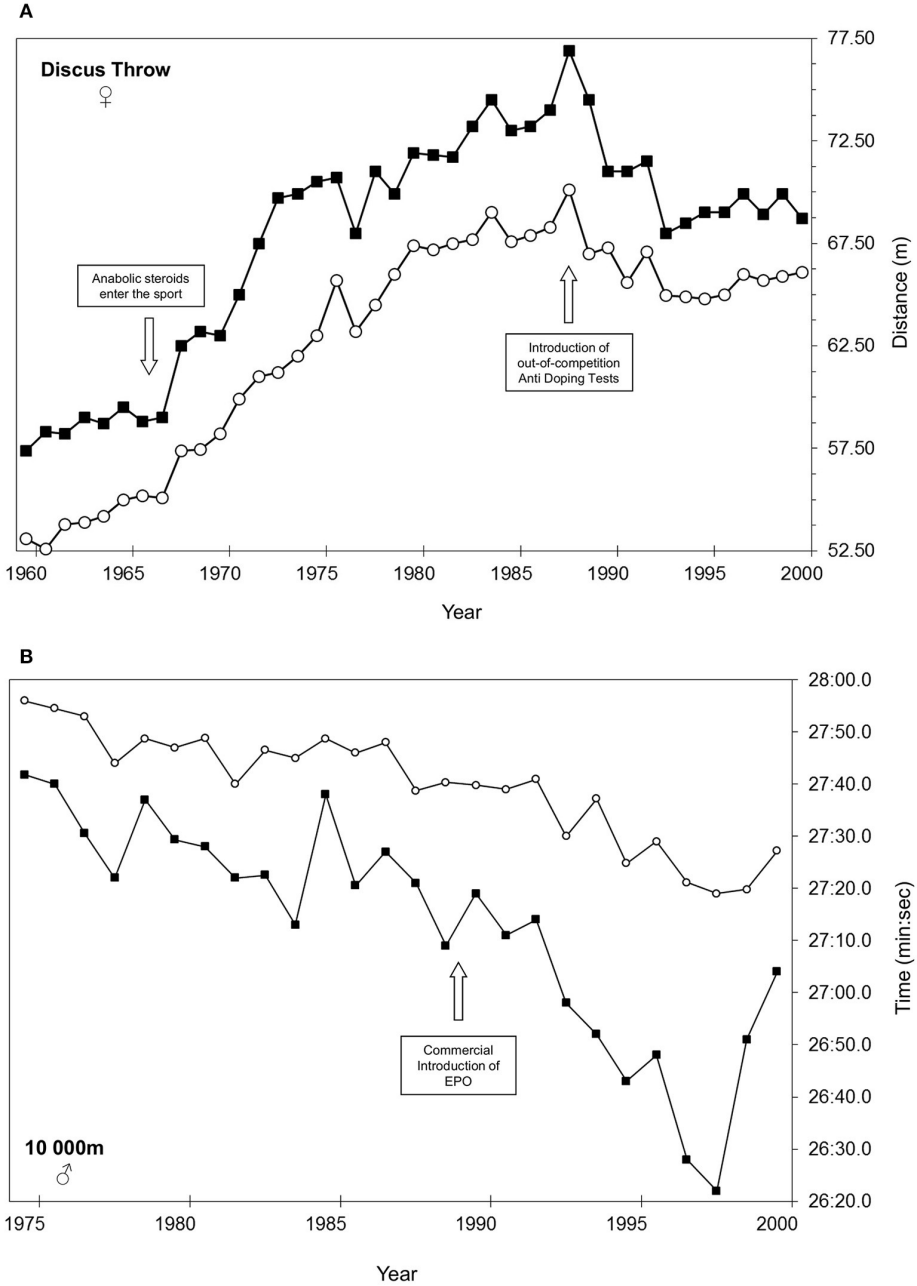
**(A)** Result of female discus throw season best performance (black squares) and the average of the Top 20 performances (open circles) for each year from 1960 to 2000 (modified from Schumacher and Pottgiesser, 2009). **(B)** Result of male 10,000 m seasons best performance (black squares) and the average of the Top 20 performances (open circles) for each year from 1975 to 2000 (modified from Schumacher and Pottgiesser, 2009).

From a more global perspective it is obvious that peak performances will increase, should a new, efficient doping agent enter the world of Sports. Classic examples include the effect of the introduction of anabolic steroids in the late 1960' on power sports (see Figure 1A, modified from Schumacher and Pottgiesser, 2009) or the effect of recombinant human erythropoietin (EPO) in the 1990 in all endurance sports (see Figure 1B, from Schumacher and Pottgiesser, 2009). Overall performance increases similar to those observed in these examples should prompt anti-doping authorities for more vigilance.

Vice versa, it is also obvious that the use of new anti-doping tests and activities could affect overall competition results “negatively,” i.e., decrease performances, as certain performance enhancing drugs will become detectable and will thus be avoided by athletes. The longevity of many world records in Track & field (many date from the 1980 to 1990) or the decrease in female discus throw performance visible in Figure 1A after the introduction of out of competition tests for anabolic steroids illustrate this point.

A more recent example is shown in Table 1. The eight best results from the national 800 m track and field championships for female runners in a country “X” were analyzed from 2008 to 2017. The distance was chosen as there are only very few confounding factors such as temperature, wind, and tactics which make results relatively comparable over years. The female category was selected for analysis due to the international dominance of the country in question that qualified several athletes for the registered testing pool of the International Association of Athletic Federations (IAAF). A competitor country was chosen for comparison.

**Table 1:** Eight hundred meters times (min:sec,dec) for the Top 8 finishers at the national championships from 2008 to 2017.

**Rank**	**2008**	**2009**	**2010**	**2011**	**2012**	**2013**	**2014**	**2015**	**2016**	**2017**
**1**	**01:54,8**	**01:57,86**	**01:59,54**	**01:56,95**	**01:57,42**	**01:59,56**	**01:59,54**	*02:01,15*	**02:00,92**	**01:58,34**
**2**	**01:56,0**	**01:58,99**	**01:59,86**	**01:56,99**	**01:57,46**	*02:00,33*	*02:00,30*	*02:01,42*	**02:01,22**	**01:59,20**
**3**	**01:56,6**	**01:59,01**	**02:00,25**	**01:57,19**	**01:57,67**	*02:00,61*	*02:00,90*	02:01,61	02:01,79	**01:59,97**
**4**	**01:56,7**	**01:59,10**	**02:00,53**	**01:58,03**	**01:57,77**	*02:00,82*	*02:01,10*	02:02,36	02:01,80	**02:00,77**
**5**	**01:58,1**	**01:59,82**	**02:00,68**	**01:58,25**	**01:58,15**	*02:01,0*	*02:01,05*	02:02,55	02:01,93	02:01,45
**6**	**01:58,3**	**01:59,85**	**02:01,31**	**01:59,08**	**01:58,55**	*02:01,01*	02:01,69	02:02,84	02:02,64	02:01,56
**7**	**01:58,0**	*02:00,16*	**02:01,64**	**01:59,86**	*02:00,06*	*02:01,18*	02:02,00	02:02,99	02:02,76	02:01,96
**8**	**01:58,6**	02:01,99	**02:02,33**	*01:59,94*	*02:00,13*	02:01,30	02:02,20	02:03,00	02:03,22	02:02,07

*Times printed in bold are performances who fulfilled A qualification standards and times in italics those who fulfilled B qualification standards for major international competitions. The results presented in normal fonts did not meet the qualification criteria for any international event*.

In our analysis we reported the yearly number of athletes who, by their results in the national championships, met the qualification criteria for major international competitions (European Championship, World Championship, Olympic Games). In Table 1, performances of the athletes who fulfilled A qualification standards are highlighted in bold and those who fulfilled B qualification standards for major international competitions are printed in italics. The results presented in normal fonts did not meet the qualification criteria for any international event. The data for this analysis were collected and verified from publicly available databases.

From Table 1, it is obvious that there is a large drop in the number of athletes fulfilling the A qualification standards for major international competitions since 2013. The first drop corresponds to the implementation of a National Anti-Doping Agency in in the country in 2009 which likely affected performance results slightly in 2010. The biggest impact is obviously the implementation of ABP by the IAAF in 2009 (Zorzoli et al., 2014), which resulted in a number of sanctions among the group of athletes from 2012 onwards (any implementation will always be seen with some delay due to the length of procedures). In absolute terms, it can be observed that there is also a large decrease in 800 m performances below 2:00 min from 2008 to 2016. It has to be noted that all reported results from the period between 2008 and 2012 come from 20 different athletes, of which 12 have since been banned for doping, mainly through the ABP. However (and worryingly), there is recently again a tendency for an increase in performance that raises the question of efficacy of the currently applied anti-doping strategy.

Similar patterns and clear relation to the implementation of new anti-doping strategies can also be seen in other middle- and long distance (1,500, 5,000, 10,000 m) disciplines in the same country (data not demonstrated) while no such findings are found in the country chosen for comparison.

In summary, such retrospective analysis of data provides an overview of the effects of new doping substances or the effect of the implementation of a new anti-doping approach on the level of performances and thus illustrates that performance profiling could be potentially used as a tool to evaluate anti-doping efficiency.

## Performance data for a forensic approach in anti-doping cases based on the athlete biological passport

The Athlete Biological Passport is a new tool in the arsenal of anti-doping strategies. It relies on the longitudinal monitoring of biological data and aims at identifying constellations in specific markers which might be caused by doping substances, rather than on direct detection of the doping substance itself (Sottas et al., 2011). When such constellations are found, the suspicious athlete is requested to explain the abnormal data. Often, the accused will present medical information which indicates a pathology which has supposedly caused the abnormal constellations. Frequently, the suspicious constellations are observed around major competitions. Thus, performance data from these competitions in the context can help to weigh the veracity of the athlete's arguments: if the athlete states that he suffered from severe pathology affecting his blood values and at the same time performs a personal best in the competition, such argument quickly loses credibility in front of any legal panel. This approach was already used with success in several ABP related cases (see for example CAS2010/A/2235 UCI v. Tadej Valjavec &OCS). It must be stressed that improvements in performance are not a sign of any wrongdoing or indication of doping *per se*, but might be considered in decision making as additional information with other reliable parameters.

## Building a framework for testing pool criteria and ensuring adequate quantities of doping tests among nations

To use their testing resources more efficiently, many national and international anti-doping organizations establish different testing pools, which classify athletes based on their performance and subsequently assign a certain amount of anti-doping resources to each category. For example, athletes in the highest testing pool are usually required to provide “whereabouts” information to facilitate out of competition doping tests, whereas for the lower testing pools, they are not. However, most national testing pool inclusion criteria are not internationally standardized, so it is entirely possible that an athlete from one country is in the highest testing pool, while an athlete with the same level of performance in another country is not even tested once. Furthermore, the amount of doping tests conducted between countries heavily varies. For example, according to the latest WADA anti-doping test statistics, an exemplary country A (1.4 million inhabitants) did 209 tests in 2015 and an exemplary country B (40 million inhabitants) did 218 tests, thus roughly the same amount despite having a 40 times larger population and thus many more athletes. This is obviously not appropriate and goes against the aim of the anti-doping community to provide a “level playing field” between nations (2).

The use of performance data to define testing pools for each discipline might be more efficient compared to the current use of the availability of resources per country. While there are certainly financial restrictions at the national level, there is the opportunity for international governing bodies to ensure such system for their disciplines and guarantee equal chances for all athletes, irrespective of their nationality.

## Other misconduct in sports

Since doping is not the only threat to the integrity of sports, performance modeling can also help to reveal cases of other misbehavior, such as match fixing or result manipulation. Similar strategies are used in gambling and Casinos worldwide already.

**Figure 2:**
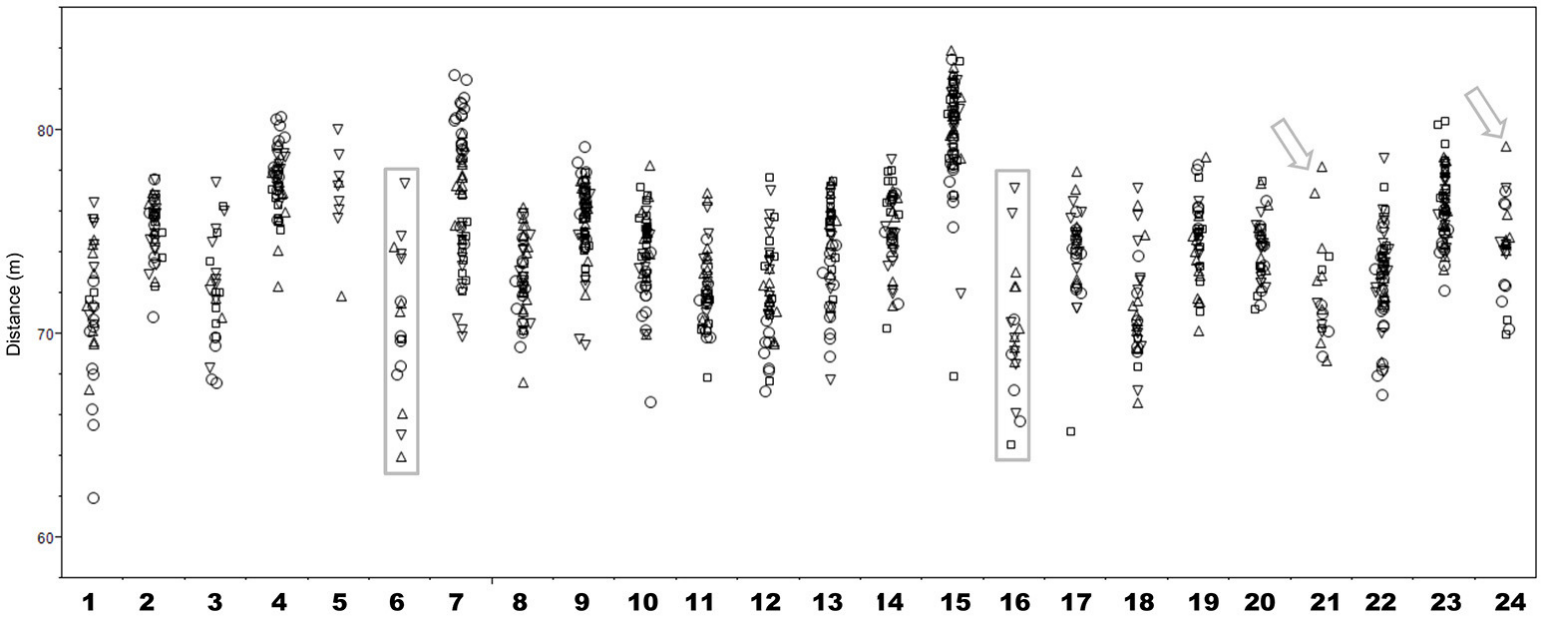
Individual hammer throw results from May to September in the years from 2014 to 2017 for 24 athletes qualified for the 2016 Olympics Games Rio de Janerio. The arrows indicate two athletes with suspicious performance in 2015 the athletes in boxes are suspected of results manipulation in 2016. The circles indicates results obtained in 2014, triangles 2015, inversed triangles 2016 and squares 2017.

As an example, we describe a case of two hammer throwers who qualified for 2016 Olympic Games in Rio de Janeiro. Figure 2 illustrates the results obtained from May to September in the seasons 2014-2017 for 24 athletes qualified for Olympic Games in 2016 in this discipline. The period from May to September was chosen as this corresponds to the peak season for the disciplines, where athletes are very likely to perform at their best. From the data, it can clearly be seen that the results are relatively stable for most athletes and range individually within ~10 m. However, a few athletes stand out: Athletes 21 and 24 achieved results much better than in previous years during the 2015 season (small arrows in Figure 2), with athlete 22 having been previously banned for doping. Thus, performance based testing would have identified these two athletes as prime testing targets, although they did not win a medal later at the Games.

More interestingly however, athletes 6 and 16 only managed to throw beyond the qualifying distance for the Olympic Games (77 m) once during the observation period (athletes in gray boxes in Figure 2). Closer scrutiny reveals that both athletes fulfilled these Olympic qualification criteria in the same competition in the same town. It is remarkable that both never competed at a level near the required qualification performance for the Olympics before or after the competition in question. The results raised suspicion and were later classified as irregular, but no further sanctions were taken since no regulation for such cases of obvious result manipulation currently exists.

This case represents even more challenges for international federations and governing sports bodies, since result manipulation demands involvement of competition officials. Thus, long-term tracking of competition results on an individual level could indeed help to identify suspicious results that would need further attention of officials or events to be investigated.

## Conclusion

These examples highlight the broad potential application of performance profiling in Sports. However, the science behind this new approach is very much in its infancy and the cases described above rely on basic empirical assessment with a very subjective component. Therefore, more research is needed to further develop this field. Main areas of interest might be the quantification of natural variability in performance in an individual and especially the development of performance over time, i.e., in the athlete's development from a young age onwards. Such data would allow a more evidence based assessment of performance changes in an athlete and move this field forward.

## Permission to reuse and copyright

Permission for Figures 1A,B reuse is acquired from Human Kinetics Inc.

## Author contributions

YS and SI: Conception of the publication; YS and SI: Preparation of the manuscript; Both authors read and approved the final manuscript.

### Conflict of interest statement

The authors declare that the research was conducted in the absence of any commercial or financial relationships that could be construed as a potential conflict of interest.

## References

[B1] de Hon O. Kuipers H. van Bottenburg M. (2015). Prevalence of doping use in elite sports: a review of numbers and methods. Sports Med. 45, 57–69. 10.1007/s40279-014-0247-x 25169441

[B2] Hopkins W. G. Hewson D. J. (2001). Variability of competitive performance of distance runners. Med. Sci. Sports Exerc. 33, 1588–1592. 10.1097/00005768-200109000-00023 11528349

[B3] Schumacher Y. O. Pottgiesser T. (2009). Performance profiling: a role for sports science in the fight against doping. Int. J. Sports Physiol. Perform. 4, 129–133. 10.1123/ijspp.4.1.129 19417234

[B4] Sottas P.-E. Robinson N. Rabin O. Saugy M. (2011). The athlete biological passport. Clin. Chem. 57, 969–976. 10.1373/clinchem.2011.162271 21596947

[B5] WADA Anti-Doping Testing Figures (2015). Available online at: https://www.wada-ama.org/sites/default/files/resources/files/2015_wada_anti-doping_testing_figures_report_0.pdf (Accessed September 28, 2017).

[B6] Zorzoli M. Pipe A. Garnier P. Y. Vouillamoz M. Dvorak J. (2014). Practical experience with the implementation of an athletes biological profile in athletics, cycling, football and swimming. Br. J. Sport Med. 48, 862–866. 10.1136/bjsports-2014-093567 24648438

